# Pre-Pandemic Social Isolation: Protection or Vulnerability in the Time of COVID?

**DOI:** 10.1093/geroni/igab046.146

**Published:** 2021-12-17

**Authors:** Lydia Li

**Affiliations:** University of Michigan, Ann Arbor, Michigan, United States

## Abstract

How do older adults at risk of social isolation before the pandemic fare during the COVID-19 outbreak? Using data from two waves (Round 9 [2019] and COVID-19 Supplement) of the National Health and Aging Trend Study (NHATS), we examined the relationship between pre-pandemic social isolation and psychological distress during the outbreak among community-living older adults (age 65+). Results show that the most socially integrated respondents had more PTSD (β=1.47, SE=.37, p<.001) and depression/anxiety (β=.34, SE=.11, p=.002) symptoms than the most isolated. Older adults who were not homebound had more PTSD (β=2.0, SE= .76, p=.01) and depression/anxiety (β=1.05, SE=.20, p<.001) than the completely homebound. With shelter-in-place and social distancing requirements, older adults who have been socially active and integrated may experience high-stress levels and may need extra support to adjust to the changes. Relatively, those who have been very isolated and homebound may experience fewer changes in their lives.

